# Selections

**Published:** 1882-02

**Authors:** 


### Selections.

---

MENSTRUATION AND ITS DERANGEMENTS. By ALFRED MEADOWS, M.D., F.R.C.P., etc., Physician to, and Lecturer on Midwifery and diseases of Women and Children at, St. Mary's Hospital.

*Menorrhagia.*—This term must be carefully distinguished from metrorrhagia, a task rendered easy by reason of the essential differences between the conditions which give rise to one and the other form of discharge. The former appears only at the regular periods at which the menstrual flow is wont to recur, whereas the latter is constantly present in patients who suffer from it. The causes tending to the production of menorrhagia are to be found in such excessive changes in the ovary as bring about a state of hyper-stimulation of the organ, and these changes may be initiated either through the nervous, or through the vascular system, or as a consequence of constitutional state. In the first case, excessive activity of the ovary results in an increase of function in the parts connected by nervous communication with it, and in these circumstances the blood supply will undergo variation commensurate with the changes set up. In such conditions of the general system as are observed in anæmia, on the other hand, fluid is easily transfused from the circulatory organs, and goes to increase the natural discharge in menorrhagic patients. In such cases constitutional treatment, by iron and other appropriate remedies, must be encouraged; but there will be also encountered varieties of this class of case, each demanding special treatment, the nature of which must be decided on the merits of each as it presents. In this connection it may be urged that no drugs are, strictly speaking, definable as emmenagogues. Some substances which, under certain circumstances, act thus, may, at another time, and under varied conditions, ensure quite opposite effects, and therefore it should be regarded as an improper license of language to retain the term emmenagogues as fixed and invariable for any particular remedy.

*Menorrhagia* may be of different types. The *congestive* variety is encountered in florid women, and where it exists the uterus will be found thickened, deeply injected, and from it will flow a bluish sanguineous discharge following the introduction of a sound. This condition will, moreover, be attended by dragging pains in the back, aggravated at the periods of menstrual flow, and which may be set down as due to a passive uterine hyperæmia. The indications for treatment of such cases point to relieving the local congestion, for which purpose leeching is probably most suitable; and it may be repeated when required. General treatment is also called for, and particularly should the liver be attended to, since it is generally found to be at fault; likewise the digestive organs. The drugs to be looked to for benefit are such as ergot, nux, etc.

In ovarian menorrhagia the symptoms encountered present well-marked features to indicate the source whence they originate. There are no signs to indicate that the uterus is directly concerned in their production; pain is felt in the groins and down the thighs, and may also be experienced in the mammæ, all these special signs of ovarian implication being absent in the form of menorrhagia previously described. In it, however, the symptoms are aggravated at the menstrual period, and find an explanation in the physiological principles already enumerated. In these cases examination of the uterus reveals no abnormality connected with that viscus, but, on the contrary, pain and tenderness are always discoverable about the region of the ovaries, and at the same time possibly enlargement of the ovary itself. Also in these cases of true menorrhagia, the menstrual act occurs more frequently than it would under normal conditions of health, and this also serves to separate these cases from those of the uterine class, in which no such increased frequency is met with. The reasons for this peculiarity again are to be found in the structural arrangements and physiological influences at work in the parts affected, and a due understanding of them will determine the nature of the treatment best to be adopted. The guide to this is in the pathology of the state, and would lead to the neglect of astringent remedies, which, from their mode of action, are improper and unscientific, since attention is to be devoted immediately to the ovaries, the seat of the morbid process that is to be attacked. The most powerful agent that can be employed in this connection is undoubtedly bromide of potassium. It exerts a direct and considerable action on the ovary, including atrophic changes in it, and thereby effects speedy diminution in the amount of discharge set up by over-excitement in its tissues. The bromide exerts specific influence over the nerves supplying the ovary, and hence the importance attaching to its use in all those instances where these nerves are in a condition of undue stimulation. Other treatment must of course be determined on the merits of each case; thus iron may be beneficially prescribed, and especially the application of anodynes locally in the form of pessaries. One of the most useful formulæ of this description is, Dr. Meadows explained, the following: codeia, 1 gr.; and atrophine, $\frac{1}{12}$ gr., in a pessary.

Menorrhagia consequent on organic changes in the uterus, may be considered as being amenable to treatment, except in those cases where such changes are due to presence of a malignant growth. The commonest form of such organic changes is so-called "sub-involution" of the uterus. This, the opposite condition to "involution," is readily understood when the sequelæ to delivery are considered. Following parturition the uterus is left in a condition of enormous hypertrophy, which, however, undergoes natural reduction, and in from three to four weeks in ordinary circumstances the organ resumes very nearly its natural condition. The process whereby this is effected is one of fatty degeneration, the uterine muscles, increase in which is that which produces the great enlargement of the organ, being thereby gradually diminished by resorption of the hypertrophied mass. The contraction of the muscular fibers themselves is a material operation, moreover, in bringing about the result; and anything which may interfere with due performance of such action will necessarily arrest the process of normal involution of the uterus, and set up a state of sub-involution, thus favoring the menorrhagic condition; and it is a matter of observation that this consequence of its existence may be present with greater frequency after abortion than when delivery takes place at the full term. In such cases, too, metrorrhagia will not rarely be discovered. The occurrences which mark this state of affairs clearly show how the discharge may be maintained during their existence; they include descent of the uterus, a consequence of its increased weight acting on the weakened supporting structures, and the increased tissue substance at the same time constitutes a much-thickened wall, while the cavity of the organ may be even double its usual length. The presence of these conditions, taken together with the history of the case, enables a positive diagnosis to be immediately arrived at. Treatment is then comparatively simple. The indications are to relieve the atony, which is the most important depraved condition, and thus restore the structures to a state in which the arrested process may once more be pursued to the natural termination.

In those instances, however, where the history given is that of a chronically inflamed hypertrophied uterus, the treatment resorted to will be different, inasmuch as the operating causes are shown to be other than in the variety just considered. In order to meet and arrest menorrhagia due to sub-involution, attempts are directed to inducing tonicity and contraction in the uterus, for which purpose such remedies as ergot, cinnamon, nux, quinia, etc., will be found serviceable. Locally, the application of galvanism, if persisted in with a determination to overcome the menorrhagia, will sooner or later be attended with relief of the symptoms, and is well worthy of most careful trial.

*Intra-uterine growths* may be either malignant or non-malignant in character, and the symptoms of their presence may include both menorrhagia and metrorrhagia. The nature of the discharges, however, may undergo very considerable alteration in accordance with changes taking place in the uterus itself. It should be carefully remembered, also, that over-frequent ovolution will give rise to excessive secretion, the discharge of which may be mistaken for evidence of a menorrhagic flow. The phenomenon of periodicity, in all such cases, will serve to arouse suspicion as to their real nature, and the further influences exerted over the discharge by, and through, the ovaries, will afford confirmatory proof concerning them.

When the fact that the uterus does contain a growth of some kind has been accurately established, then, if it be malignant in character, further decision respecting its special nature will be materially assisted by the signs its presence affords. Thus, the situation and character of the pain experienced by the patient will be a valuable means of diagnosis in cancer of the cervix, scirrhus. Absence of painful sensations is by no means of uncommon occurrence, whereas in that description of cancer which is found invading the fundus uteri agonizing pain is an all but invariable accompaniment, and it is never unattended by severe attacks of pain. The knowledge already gained of the nerve distribution of the uterus will render the explanation of this difference a simple matter, as also it will indicate why operation on the neck of the womb may be resorted to without the fear of unduly disturbing the patient.

There is also a distinct connection between the pain experienced and the amount of discharge from the organ. Thus the greater the degree of pain the smaller is the flow; and the more considerable the discharge the less excruciating is the pain, or the greater, in other words, the freedom from pain. This correlation is of immense value as a means of diagnosis in case of cancer uteri, and will often become a guide in the question of operative procedure. Whenever a copious discharge is met with in patients who suffer but little, and in whom the presence of a cancerous growth has been made out, then the conclusion is justified that the cervix is the part implicated, and never the fundus of the uterus. Again, as to the appearance of the discharge. This will be of a dirty fœtid nature, only in cases of malignant tumors, unless it be that it arises from a sloughing sore, or from pent-up secretions. In malignant cases it is always present.

The presence and nature of the growths under consideration need to be made out with care and certainty ere their treatment can be hopefully resorted to; and for this purpose several modes of investigating them must be pursued. Several aids to physical examination can be employed, and the chief may be noticed in succession. The fingers of the operator are the most important of all these aids ; by them the size, consistence, etc., of the cervix are at once determined ; they unerringly ascertain the presence of growths on the walls they are in contact with, and as well, too, they will enable a determination to be arrived at concerning the preservation of the tumor, whether that is to be properly associated with one or other of the groups of cancerous growths, or is of non-malignant origin, as, *e. g.*, polypi, etc.

At this point Dr. Meadows proceeded to describe at some considerable length the numerous tumors to be found in the uterus under abnormal conditions, and the way in which they would be found productive of menorrhagia. From this subject he then passed to a consideration of the second most important aid to physical examination, the *uterine sound*, which he likened to an elongated finger, by the employment of which valuable information might be gained concerning the length and extent of the uterine cavity, the situation and nature of growths in its walls, etc., etc. He next enumerated the various kinds of tents, sponge, tangle, etc., also of service as aids to examination, which acted by dilating the canal of the cervix, and so admitting freer inspection of the interior of the womb.

In respect of *interstitial tumors* of the uterus, Dr. Meadows strongly inclined to the view that *enucleation* is the most appropriate treatment to adopt toward them ; and he expressed his conviction that in the future it would be universally employed. In the majority of cases the tumor is, he explained, so free of the proper tissue of the organ as to admit of being safely everted, and he had been surprised sometimes to find how thin the wall could be which permitted this to be done with absolute safety.—*Medical Press and Circular.*

---

### HEATON'S OPERATION FOR THE RADICAL CURE OF HERNIA.*

By GEORGE W. GAY, M.D., Surgeon to the Boston City Hospital.

The Heaton method of treating inguinal hernia for a radical

* Read before the surgical section of the Suffolk District Medical Society, November 19, 1881.

cure consists in moistening the fibrous tissues of the inguinal canal and rings with a preparation of white oak bark, and applying pressure by means of a compress and bandage to keep the canal closed, and prevent the descent of the hernia till the contracted tissues become strong enough to support the strain imposed upon them. The originator of the treatment claimed that by this method of "tendinous irritation," a permanent contraction of the fibrous structures was produced, which resulted in a lasting cure of the affection.

The fluid recommended for injection is composed of fourteen grains of the solid extract of white oak bark, thoroughly rubbed up with half an ounce of the fluid extract of the same drug by the aid of gentle heat. The mixture is thick and muddy, and requires thorough shaking before using.

The operation is performed as follows: The hernia having been reduced, and the sac also, if possible, an instrument resembling the hypodermic syringe, charged with the astringent, is thrust directly down through the skin into the external abdominal ring, and the point of the needle carried up the inguinal canal in front of the spermatic cord to the internal ring. The fluid is deposited slowly while withdrawing the instrument, the point of which is to be moved about in all directions, in order that the astringent may be distributed as evenly as possible throughout the canal. A compress and bandage are applied at once, and worn for a few weeks, when, in the successful cases, the rupture is cured, and requires no further support.

Twenty-four hours after the operation there is usually present some effusion and tenderness in the inguinal region at the seat of injection. The former may remain for an indefinite period; the latter commonly subsides in a few days, except in those unfortunate cases which terminate in suppuration. In some instances no thickening or effusion can be detected after the operation, while in other cases complete absorption of the exudation takes place after a time, and the parts return to their former condition. At the end of a fortnight, in the favorable cases, when the patient is allowed to leave the bed, the external ring will be found much reduced in size, and it is with difficulty that the finger can be introduced into the canal, where, previous to the operation, it passed easily.

Should any of the fluid be allowed to escape into the areolar structure outside the canal, it is apt to produce a mass of induration in the loose tissues, which may persist for some time, but which, from its location, size, mobility and gradual absorption, can seldom serve in any degree to prevent the hernia from coming down. Furthermore, cellulitis and abscess may result from this cause. Suppuration occurred in two of our patients. The abscesses were deep-seated, apparently extending through the anterior wall of the inguinal canal. The resulting infiltration entirely disappeared in a few weeks after the abscesses closed, and in one case the hernia returned. These complications increase the suffering, prolong convalescence, and, in our opinion, add nothing to the success of the operation.

Dr. Davenport, who edited Dr. Heaton's book, and who was a careful and conscientious observer, always strongly insisted to the writer upon the importance of setting up only a very moderate local action by the injection; anything like severe inflammation was to be avoided if possible. He believed that white oak bark had a mild yet persistent astringent effect upon fibrous tissues, which was more lasting and less violent than that resulting from ordinary inflammation. Hence, it is the fibrous, and not the cellular structures which require the application of the astringent.

Where is the fluid deposited, in this operation?

This is an inquiry that has often been made, but to which, in the absence of any examination upon the cadaver, it is difficult to give a satisfactory answer. Theoretically, it should bathe the fibrous structures in contact with the neck of the sac. Practically, the surgeon, depending largely upon his anatomical knowledge of the parts, carries the needle up to the vicinity of the internal ring, and slowly empties the syringe during its withdrawal. Whether the sac is penetrated or not is probably largely a matter of luck, rather than of skill, for in cases of old hernia it would seem hardly practicable to guide a needle between the sac and walls of the canal without penetrating one or the other. In some cases the needle moves about in the canal with great freedom, giving one the impression that it has entered the cavity of the peritoneum; yet in twenty-seven operations I have never seen anything like peritonitis. With few exceptions, there has been only a moderate local disturbance, which did not require treatment, and which subsided in a few days. Although the pain of the operation is not severe, yet in children an anæsthetic is required to prevent the struggling and straining from forcing out the contents of the rupture before the bandage is applied; timid and nervous people also need it to enable them to keep still. Opiates are not usually called for during the after-treatment.

Below will be found a brief report of *all* the cases operated upon by me, both in hospital and private practice, with the exception of two, which have been under treatment but a few weeks. The results are stated as fully as possible, and no patient is reported *cured* who has not remained well for at least a year after discarding all support to the rupture.

*Case* I.—A waiter, aged twenty-four years, entered the hospital suffering from an inguinal hernia of moderate size on the left side; a truss had been worn. September 9, 1877, the patient was etherized, rupture reduced, and ten drops of the Heaton mixture injected into the inguinal canal. A compress and bandage were applied, and the patient remained in bed two weeks. He was discharged, wearing a bandage, at the end of five weeks. The external ring had been reduced to one-half its former size by the operation, and the rupture had not returned.

*Case* II.—A sailor, thirty-four years of age, with a reducible inguinal rupture of seven months' duration, was operated on at the same time, and in the same manner as the previous case. He was discharged in four weeks, wearing a truss; the ring was contracted somewhat, but not sufficiently to control the hernia.

*Case* III.—A harness-maker, aged forty-five, subject to asthma and bronchitis, had a right inguinal hernia of some years' duration; the ring was large, and rupture uncontrolled by a truss. Operation June 10, 1878. Although the size of the ring was somewhat reduced, the intestine came down during an attack of severe coughing, and he was discharged not relieved.

*Case* IV.—A policeman had an omental rupture of two years' duration, which extended half-way to the bottom of the scrotum, and could not be controlled by a truss. The operation by injection was performed twice in as many months, with the result of reducing the ring to the size of a pipe stem, but a strand of the omentum still came down, though it could easily be kept up by a truss. Being very anxious to obtain a radical cure, he afterward submitted to an operation for the removal of the protruding omentum, but with only a partial success. He is now wearing a truss.

*Case* V.—A boy, a year and a half old, with a double inguinal hernia, was operated upon in 1878, in the out-patient department of the hospital. The after-treatment was neglected, as the mother failed to bring him back to have the bandage readjusted. The operation did no good, and the child now wears a truss.

*Case* VI.—James, twenty-one months old, right inguinal hernia; injected with three drops of the oak bark; wore bandage only a week; remained well at the end of fourteen months.

*Case* VII.—Michael, two and a half years of age, had a right inguinal hernia which had lasted six months; the ring was large, and two operations were performed. Three years later the rupture came down, only after great straining; he had worn no support.* It seems fair to consider him much relieved by the injection.

*Case* VIII.—A house painter, aged twenty-four, subject to convulsions, was injected for an inguinal rupture. The fits returned three days after the operation, when he jumped out of bed, pulled off his bandage, and the rupture returned. No benefit was received from the operation.

*Case* IX.—Daniel, aged twelve, a thin, spare boy, had a scrotal hernia with large rings, and loose pillars. Was injected twice within two months. He remains well at the present time, and has not worn a support for nearly two years.

*Case* X.—A boy, four years of age, was operated upon for an omental hernia running into the scrotum, with little if any relief.

*Case* XI.—John, six years old; inguinal rupture, which had come down only a few times; it was reduced under ether about a week before the operation by injection. The ring was small, and hence peculiarly adapted to this operation. The little fellow

* I have lately repeated the operation upon this child.

was running about in a week, and has had no return of his rupture. It is now nearly two years since the operation. This patient would very probably have recovered by wearing a well-fitting truss.

*Case* XII.—For a year Miss ——, aged thirty-eight years, had suffered a good deal from an omental hernia, which came down into the right labium in spite of any truss she could apply. The operation was performed as in the male, and she remained in bed about a month. The resulting inflammation in this, as in all the cases narrated thus far, was very moderate, never threatening suppuration. She wore an abdominal supporter for several weeks. Fourteen months after the operation she remained well.

*Case* XIII.—A man, fifty-eight years of age, entered the hospital, suffering from the early symptoms of strangulation of an inguinal hernia. It was reduced under ether, and the operation for a radical cure performed some weeks later. When he left the hospital, at the end of three weeks, the hernia had not returned, and the little finger could not be passed into the external ring. As the rupture was intestinal, the operation promises to be successful.

*Case* XIV.—A baby, ten months old, was brought to me with double inguinal hernia, which trusses did not keep in place. Both were operated upon for the first time over a year ago; as the child has since had measles and whooping-cough, without bringing down the left rupture, we may safely call that one cured. Suppuration followed the injection upon the right side, and the operation has been repeated twice without success. The rings are large, the inguinal canal is short, and thus far no truss has been found to control the hernia. The operation is to be repeated after the child has fully recovered from whooping-cough.

*Case* XV.—Mr. F., aged twenty-three, suffered a good deal from inguinal herniæ, which could not be controlled by trusses. The rings were large, pillars thin, and abdomen flat. Both ruptures were operated upon without ether a year ago. Large effusion followed the injection, resulting in an abscess on each side, which was a long time in closing. Neither hernia has returned; the patient wears a double truss, and has been greatly relieved by the operation.

RECAPITULATION.

Number of patients, fifteen; cured, four; relieved, eight; not relieved, three. Number of ruptures, eighteen; cured, five; relieved, eight; not relieved, five. Number of operations, twenty-three.

With two exceptions, the above cases seemed to be favorable ones for the operation. That it was not more successful may very likely be due, in part, to the lack of skill and experience in the operator; in part to the imperfect after-treatment, especially in several instances, occurring in the out-patient department of the hospital; and finally to the fact that a second operation could not be obtained in several cases, in which not quite sufficient contraction resulted from the first.

While I do not feel justified, from my limited experience, extending over only four years, in expressing a decided opinion as to the real value of the oak bark treatment of hernia, yet my impression is, that in the inguinal variety of the affection it is often a good method, and is worthy of further trial and study at the hands of competent surgeons. It would seem peculiarly adapted to cases with small rings, and to cases occurring in children, when Nature herself is making a constant effort to correct the deformity, and requires but little assistance to enable her to accomplish the result.

I know nothing of the merits of the operation in other kinds of hernia; but in the one under consideration I can but conclude that it is safe; it is not very painful; it is not very difficult to perform; it does little harm, even if it does no good; it will cure a certain number, and will relieve others.—*Boston Med. and Surg. Journal.*

---

NERVE STRETCHING IN TETANUS—SUCCESSFUL. By W. I. WHEELER, M.D., T.C.D. F.R.C.S.I., Surgeon and Lecturer on Clinical and Operative Surgery to the City of Dublin Hospital; Member of Council Royal College of Surgeons.

The operation of nerve stretching has, of late years, been often practiced, and has been a subject of much interest to, and investigation for, most practical surgeons, although the case I am about to record, viz., nerve stretching in acute traumatic tetanus, scarcely as yet comes within the present literature of the operation, only one case, as far as I know, being fully and well recorded of nerve extension in tetanus. I feel that a limited epitome of one or two of the earlier accounts will render this communication more interesting. Truly, many cases recorded in times past bear on the operation of stretching nerves, although a different explanation is given, to what now, they would seem to require ; this is well exampled by a case of secondary amputation performed by Mr. Langstaff, for a neuralgic stump, in which he states he drew out the nerves before dividing them, and that the neuralgia was cured. He performed this operation in order that the nerves might be free from cicatricial tissue, but it is reasonable to assume that the favorable result was due to the *nerve stretching.*

Amongst the earliest operations of this kind we find the following remarkable case, published by Professor Nausbaum (*Deutsche Zeit. f. Chirurgie*) which I will briefly record:

A soldier, æt. 23, received a blow with the butt end of a rifle, on the nape of the neck, and another on the left elbow. At the former place an abscess formed and was opened and duly healed up again. He afterward suffered from contraction of the left pectoral region, the whole left upper and fore-arm, and hand of the same side. This contraction was a spasm, which became more violent the more attempts were made to oppose it ; besides, there extended over all the dorsal aspect of the fore-arm an anæsthesia, which increased to such a degree that deep incisions, tenotomies, etc., could be practiced without any pain to the patient. The spasms varied in intensity, and were so violent that for hours the finger tips were driven into the palm of the hand ; the great pectoral muscle was always as hard as stone. Numerous were the remedies applied—iodine, mercury, belladonna, and the constant and interrupted galvanic currents, etc., etc., without affording the slightest relief. Professor Nausbaum operated after this manner :

The patient being under chloroform, an incision about three inches long was made over the site of the ulnar nerve, which was lifted, gently extended, and then replaced. The wound was cleansed, and closed by suture. A second incision was made in the axilla over the artery. The nerves, which closely surround the vessel, cutaneous as well as muscular, were drawn out one by one, the median, radial and ulnar being recognized to a certainty by the twitching of the muscles of the finger, which they respectively supplied, and after being subjected to the same treatment as the ulnar nerve, were replaced and the wound brought together; lastly, an incision was made over the left clavicle, such as would suit the ligation of the sub-clavian, the platysma having been divided, the four lower curved nerves were isolated, traction was exercised upon each, and each was followed with the tip of the right forefinger to its point of exit from the vertebral column, here each was pushed upward and downward, and right and left. A good tug at the nerve in a direction, although it were to be pulled away from the spinal cord, completed the operation. The four upper curved nerves were not touched, as there had been no indication that there was any lesion of the phrenic nerve at its origin. The operation was successful beyond expectation, the spasms did not return, the forearm and fingers could be flexed and extended at will, and the skin of the arm, which before the operation was so devoid of sensation that puncture and the application of hot sealing wax were painless, had regained sensation to such an extent that the patient, even when blindfolded, could localize the slightest touch. The man made a good recovery, although he had of necessity to run the gauntlet of pyæmia, nearly always present, then rife in Nausbaum's hospital.

Close after Nausbaum, we have cases recorded by Billroth and the late Mr. Callender. The observations of the latter gentleman, well worth perusal, may be found in the Clinical Societies "transactions;" were it within the province of this communication, I could myself relate cases with satisfactory results from stretching the supra-orbital, facial, spinal accessory, and external poplitial nerve, and quote published statements, that substantial and advantageous results have followed the stretching of the ischiatic nerve in Duchenne's paralysis. That relief may be obtained in such cases is quite tenable, but the pathology of the disease negatives the assertion that more than that can be gained; the notes of the following case under my care were chiefly taken by the house surgeon :

Teresa M., æt. 8, a strong, healthy child, was admitted to the City of Dublin Hospital on the 10th of October, 1881, suffering from what she stated to be a sore finger. Eight days previous to admission a stone fell on the middle finger of her right hand, bruising it severely ; the finger had been dressed and placed on a splint by the medical man who saw the case, but, owing to the neglect of the parents, the dressing had not been changed for six days previous to admission. On examination the end of the finger was found to have been destroyed by gangrene, and fell off with the foul dressings, leaving the end of a fractured phalanx (the second) protruding; this fell away at the joint next day. The patient did not complain of any pain in the hand, but did of pain in the back, which was stated to be caused by a fall off a table. When the question of making a neater stump was under consideration, it was noticed that the patient lay in a peculiarly stiff way in bed ; operative procedure was postponed, commencing tetanus was diagnosed, and was followed by the train of usual symptoms. The muscles of the back and abdomen became rigid, the sterno-cleido-mastoid and trapesic muscles were also in a state of rigidity, the risus sardonicus was well marked, and strong tetanic spasms backward (opisthotonos) contorted the child's body; the spasms came about every two hours. The treatment was locally, opiate stupes applied over the wounded finger, enveloping the hand as far as the wrist, and ice to the spine and head. Internally, bromide of potassium, belladonna, and cannabis indica were administered; chloral hydrate was given by the rectum to produce sleep; the nourishment was chicken broth, milk, and beef-tea. The room was carpeted, and a screen placed round her bed. There being no improvement from the above treatment, but, on the contrary, the patient getting worse, and the spasms increasing in intensity, on Sunday morning, the 15th October, I determined to stretch the median nerve in the fore-arm. The child being put under the influence of chloroform (my colleagues, Dr. H. Benson and Mr. Butcher, were present), I made an incision about two and a half inches long in the forearm and quickly caught the median, closely applied to the posterior surface of the flexor digitorum sublimis, which appeared somewhat red. I stretched the nerve between two fixed points, and also pulled it firmly downward toward the wrist joint in its long axis; while under the chloroform the tetanic spasms stopped. The wound was brought together by means of strips of American rubber plaster, and quickly healed. No deleterious carbolic spray was used, but there was minute attention paid to surgical cleanliness before bringing the edges together. For two hours after the operation the spasms increased a little in frequency, but gradually became less frequent. Never were they so intense from the time of the operation, and from this time the recti muscles of the abdomen became relaxed. The temperature was always normal or sub-normal,* and the pulse ranged from 60 to 70 per minute, save on two occasions when it was 110 and 90 per minute. The further history of the case was a gradual progress toward recovery; the last recorded twitch, for it could not be called a spasm, was on the 20th of October. The patient is now quite well and she has returned to the country. In her photograph (here produced) can be seen, on close examination, the line of incision. On testing the sensation in her right hand with the æsthesiometer, three days before leaving the hospital, it was found unimpaired, as also motion.

As before stated, the only well recorded case of nerve stretching in tetanus that I can find is that reported by Dr. Paul Vogt, who cut down on the brachial plexus, and pulled it in a case of traumatic tetanus occurring fifteen days after an accident which inflicted wounds on the palmar and dorsal aspects of the same hand. He states that there were attacks of the most powerful opisthotonos, that there was no pain in the forearm or arm, not even tenderness, and that the wounds were healthy and granulating. Slight spasms occurred in his case on only two occasions after the operation, but there was some stiffness of the jaws remaining. In discussing the treatment of *traumatic* tetanus, it can be done under two heads, viz., by operative measures, and medicinal measures. The results of treatment are so unsatisfactory as to justify any reasonable line of action that has a chance

* The highest temperature recorded in tetanus is that by Wunderlich. Even the thermometer stood at 112 Faht., and the temperature continued to rise for an hour after the cessation of all signs of life.

of success. Under medicinal agents we could not well be at a loss, as almost every medicine in the Pharmacopœia has, some time or other, been prescribed for this affection. On the contrary, operative measures are much more limited. I may name amputation, actual cautery, division of nerve trunks, and nerve stretching; of these I selected the latter, believing it to give the best chance to my patient. That the pulling and pulling forcibly of a nerve is not followed by any untoward results is manifest not only from the cases I have quoted, but from those recorded by Billroth and others, where large nerves have been exposed and roughly handled, neither was their motor power nor their nutrition impaired; but when we come to consider how the stretching of a nerve relieves pain, how it stops tetanic spasms arising from central irritation, with our limited physiological knowledge of the spinal cord it is difficult to offer an explanation.

Now Flaubeit and Le Bret have shown that inflammation of the spinal cord may follow as a consequence of irritation of the brachial plexus, and Brown-Séquard has confirmed this, and illustrated the extension of irritation through a nerve trunk to the spinal cord; developing symptoms through the extension of the mischief in the nerve centers, to parts removed from those first involved, hence, the necessity for the recognition and discerning between a peripheral and a central irritation. Nausbaum has made a speculative statement, that in neuralgia the stretching is of service by altering the relations of the nerve fibers and improving their nutrition, but this would not account for the cessation of spasms due to tetanus. It would seem that as neither sensation or motion, even in violent nerve stretching, is impaired, that it must be a temporary numbing of the nerve, by which numbing there is a cessation of the power of the nerve fibers to transmit abnormal impressions, and thus the centers have time to resume their normal condition and control. *Apropos* of this, Dr. Brown-Séquard has proved that if a nerve is exposed and frequently washed with ether it will be unable to transmit any irritation. But, it may be asked, will not the division of the nerve break the chain of these abnormal impressions? I think experience teaches us the contrary, for if there is a neuritis present, as I believe there was in my case, the limits of the affection are unknown, and in the cases recorded by Drs. Sands and Seguin, in which portions were cut out from the brachial plexus, the nerves of which were found matted together, the patient was only relieved, as the neuritis probably extended further on the central side. Knowing these facts, I was prepared to stretch the brachial plexus of nerves, were 1 not convinced as I was by the relaxing of the abdominal recti, and afterward by the lessening of the spasms, both in frequency and intensity, that the amount of stretching I practiced would be sufficient; but, in thinking over the case, it has occurred to me that had I stretched the brachial plexus, like Dr. Paul Vogt, the spasms might have ceased almost immediately. I deem this case one of much interest, and being anxious to know if there had been any cases of nerve stretching for tetanus in the sister Island that I could quote here to-night, I inquired of Mr. Bryant, who informs me he cannot give me reference to any case of nerve stretching for tetanus in London.

---

### RABIES—A POSSIBLE CAUSE AND A PROBABLE PREVENTIVE.

By L. L. DORR, M.D., San Francisco.

It is probably owing to the great obscurity that surrounds the etiology, pathology and treatment of rabies, that it is exciting so much study. Taking these divisions of the subject up inversely, it may be remarked that experience in the treatment of this terrible disease has thus far furnished no favorable results. In this respect there has been less accomplished than in the case of any disease known to both ancient and modern times. In the few cases chronicled as cured there have invariably been good grounds to question the diagnosis; for in a perhaps purely nervous disease like this, there is plenty of room for exercising the imagination both of the patient and of the physician. It is safe to say that in no case of undoubted rabies has death failed to result speedily, and that there is no known cure.

Of the pathology, we have none that is recognized by the general profession. Whether it is primarily a disease of the blood, which sooner or later affects the nervous system, or whether the effects are exerted directly on the nervous system, is not known. Both systems seem to be the theater of the disease in man. No contusion or rupture of the skin can occur but there is of course more or less of an impression on the nerves ; but it seems to be necessary that the poisonous saliva shall reach the absorbents to produce the effects, and then it is a comparatively few who are bitten by a really rabid animal who take the disease. Injury of the peripheral nerves when there is no possibility of the wound being poisoned, may produce tetanus, a disease much resembling rabies. A comparison of these two diseases shows that the history is similar, the symptoms being markedly alike in many respects, and the results of treatment in most instances not much different, for Dr. O'Beirne, of Dublin, witnessed two hundred cases of tetanus without a single recovery. It may be that rabies is a form of tetanus, but that the special poison has a particular affinity for the nerves governing the muscles connected with the acts of deglutition and respiration. There are poisons that produce analogous, peculiar and special effects. Dr. A. Flint, Jr., says : "Woorara, a poison, has the remarkable property of paralyzing the motor nerves, but leaving the nerves of sensation intact." This drug has been used in the treatment of both tetanus and rabies for these reasons, but with no marked results. Cantharis has a special effect on the mucous membranes of most persons, and it may kill if the dose is sufficiently large. There are many drugs, of vegetable and mineral origin, that have special effects on certain organs.

The symptoms of rabies are well-known, and generally unmistakable to an unprejudiced mind ; but until we more fully understand the causes, we cannot expect to prevent this malady. The same may be said of the pathology ; for until we understand the pathology, especially during its incubation, we cannot expect to treat the complaint intelligently or successfully. But little has been said of the etiology in all that has been published of late on this subject, and when the pathology and treatment have reached a non-progressive stage, it is time—not to abandon them—but to direct the attention of thinking people to the cause, that steps may be taken for prevention ; and there is something to be said on this point. We are not satisfied to sit down and say, "What can not be cured must be endured;" for what can not be cured in medicine must be prevented, says modern science. In recent medicine, it is possible that the prevention of disease has done more to prolong human life than therapeutics, whether based on pathology or not. It may be that such will be the case with rabies; and that in the absence of any cure, and with certain death in prospect, the grain of prevention may be of great value. As instances where progress has been made by adopting this line of defense, it is only necessary to mention the management to-day of yellow fever, cholera, small-pox, etc. For these infectio-contagious diseases there are no cures, but we see how much can be accomplished by prevention; which, in fact, is almost total annihilation. How can these arguments be applied to rabies?

*The Possible Cause.*—In a study of the natural history of the dog, we find that he is an animal existing in nearly every country of the world, both in the wild and in the domesticated condition. He is indigenous to nearly all lands. The theory that the dog is a descendant from the wolf is not tenable.

Rabies has been known among dogs as far back as history speaks of them. It is also found that it has prevailed most in the most thickly-settled countries, and those having the most intimate and constant communication with other nations; and from those peoples it can be traced just in proportion as they made discoveries and conquests of other lands; for wherever civilized man has gone he has taken dogs, and in those lands rabies has then appeared, and not until then.

In the Mauritius Islands it was not known until 1821, when it appeared soon after a dog arrived there on an English ship from Bengal. It is not mentioned whether this dog was allowed intercourse with the native dogs, thereby giving opportunity for generating or spreading the disease by contagion, but it is presumed the greatest liberty existed.

In Labrador, Liberia, Australia, Van Diemen's Land and New Zealand, places in but comparatively slight communication with the rest of the world, rabies is seldom if ever heard of. In relation to Greenland, heretofore reckoned in the list, Professor Agnew says, in his recent "Surgery:" "In 1863 it prevailed to such an extent in Northern Greenland as to destroy all the dogs in certain localities." It must be remembered that communication with Greenland is getting more and more frequent, and that the Esquimau dog is said to be fast crossing with the wolf. Erichsen says: "Rabies is not known in Central Africa in any animals."

As regards China and Japan, countries notably exclusive of both men and animals from foreign lands, and where they have mostly one breed of dogs, rabies is seldom heard of. Authorities differ, however, in this respect. Dr. H. W. Boone, surgeon in charge of St. John's College, Shanghae, China, writing under date of June 28, 1881, says: "The ordinary Chinese dog is large, sort of half-wolf, like Indian or Esquimau dogs, but they have small Pekin pugs and small Canton dogs. Chinese dogs occasionally cross with European dogs, but only at the few open ports. The pure Chinese dogs have hydrophobia, and did so in early days, before foreign contact was common. Japan the same." Dr. D. B. Simmons, for many years a resident in Yokohama, Japan, writing under date of March 10, 1881, says: "I have never seen an unmistakable case of rabies in a Japanese. I saw one case in a foreign child who was bitten by a foreign dog, a Newfoundland. The dogs here are large, wolf-like beasts, having their origin, no doubt, in China."

Without doubt, dogs have been taken to all of these places, and perhaps allowed to live there for years, but they were probably dogs of a pure and original breed, and were not permitted to accompany or cohabit with the native dogs. And here hangs the point of my argument as regards the cause of rabies—that is, that a dog of a pure and original breed is incapable of generating rabies, and that it never appears in any land until such dogs breed in-and-in with dogs of other countries, producing the mixed or mongrel dog. All dogs receive and give the contagion, but only the mongrel can *generate* it. Such seems to be the proper deduction to make from the history of rabies, and such is the conclusion that observers of dogs and of this disease are fast approaching. Charles Hamilton Smith says that "the Newfoundland dog is an original breed, and true hydrophobia does not attack them in their native land." The same author, in speaking of Arctic dogs, says: "Arctic dogs in their native regions are not liable to canine madness." He also says: "Of all domestic dogs, the greyhound is the least liable to hydrophobia." It is also true that the greyhound is the least apt to breed with other dogs; they are peaceable, keep mainly by themselves, and are no doubt of a pure and original breed. Erichsen says: "Among dogs, rabies is the most common in those of mongrel breed, seldom affecting those of pure blood." John W. Hill, R.C.V.S., in speaking of the causes, says: "I am very much inclined to think breeding in-and-in encourages its development." Dr. Verity, in the Manchester *Courier* of 1876, says: "I have always had a strong opinion that breeding in-and-in tends to produce hydrophobia in the dog." There are many instances in human beings where crossing the race tends to produce men inferior in intellectual force and physical bearing, with a strong tendency to disease. If we want a perfect specimen of the intellectual and physical make of any race, we find it among those of pure and unadulterated blood. The crossing of nations of the same race produces, undoubtedly, a superior class of men, but the crossing of different races only produces inferiors, natural criminals and idiots. There are a few exceptions, but they only make the rule more apparent and forcible. The Caucasian and Indian crossing we all know the result of—weak, criminal, and sickly offspring. There are no King Philips or Sitting Bulls who are half-breeds. The results of the crossing of the negro and white man are numerous among us, and generally they are objects of pity, and die early. The few with us of the cross of Chinese and Caucasian are the despised of both nations. The Mexicans are largely a cross between the Spaniard and the native Indian, and are a weak, degenerating, dying people, far lower in intelligence and physique than the Spaniard of Spain or the historical Aztec. To return to the brute creation, it is only necessary to say that the breed is not improved by crossing the common horse with the Arabian or Shetland. Cattle are not improved by crossing with the buffalo. These instances all seem to admonish us to keep the breeds, whether of human beings or animals, pure and of the original race. It may be that rabies is the result of the violation of this seeming law of nature; and if so, the prevention would be easy —that is, to allow none but dogs of a pure and original breed to live. How easy this would be to try in a land like England, where laws are made to be enforced, and it would not be hard to determine which were of a pure breed. It is possible, to my belief, that if dogs of different breed were prevented from crossing, or their progeny disposed of, rabies might be prevented. In the absence of a cure, the trial of this measure may be deemed advisable.

The question naturally arises, Which are the dogs of a pure breed? We have to regret that nothing is *positively* known on this point, history being incomplete. Recent authorities, such as Charles H. Smith, Thomas Bell, and Youatt, speak in no decided words on this point, using the indefinite terms probably, evidently, etc.; but the following-named are with little doubt directly descended from the wild dog: the shepherd dog, the mastiff, the greyhound, the hound, the spaniel, the Newfoundland, the Esquimau, the terrier and the cur. The shepherd has been considered the primitive dog. He is no doubt the original dog of Western Asia and Egypt. The mastiff may be indigenous to England or the higher lands of Asia and Africa. The greyhound is very ancient, having been known three thousand years ago, when it differed in no important particular from that of the present day. The British Museum contains a group of greyhound puppies in stone, from the ruins of the villa of Antonius. The greyhound was an inmate of Anglo-Saxon kennels in the time of King Elfric. The hound and spaniel are natives of Spain. The Newfoundland is indigenous to that island, and was not known in Europe until imported from there. The Esquimau dog is a native of the more northern latitudes, and is more like the wolf than any other species, yet is reckoned as the native dog. The majority of those seen at the present time may be a cross with the wolf. The terrier is considered indigenous to Great Britain. Curs are the most numerous, and are the native dogs of many countries; but, having no well-marked characteristics, they have received no definite name, and are often confounded with the mongrel dogs. There may be others, but these are the principal ones.

*The Probable Prevention.*—It has been very generally admitted that climate makes no difference with the appearance of the disease. Professor G. Canettoli, in *Lo Sperimentale* for June, 1875, says, in writing of rabies: "This is a disease of all climates; the extremes have the smallest number." Mr. Hill says: "The influence of climate, season, or sex would appear to have little bearing on the subject." This conclusion is from a European standpoint.

The Pacific coast of America presents some peculiar features as regards this disease. We have in this city, and in a portion of the State, a peculiar climate, which cannot be compared with that of any other portion of the world. But we have also in this large State, and on the Pacific coast, thickly-settled towns and cities where the extremes of heat and cold are well marked, and can be brought into comparison with those of places in the Eastern States and Europe; yet, from Behring's Strait to Cape Horn, not a well authenticated case of rabies was ever known. Dr. Logan, recently United States Minister to Chili, in a little work published in Chicago, called "Physics of the Infectious Diseases," says: "Of the remarkable disease called hydrophobia, the author feels himself justified in saying that it has never been known upon the whole coast." Dr. F. C. Valentine, of Guatemala, writes, under a recent date, and after eleven years' residence in Central America: "I have not encountered one authenticated case of rabies during my residence here." Dr. G. Chismore, of San Francisco, a gentleman fond of dogs, and who lived several years in Alaska, says, under date of June 11, 1881: "I never saw or heard of a case of rabies among Alaskan dogs." Dr. F. W. Hatch, permanent Secretary of the State Board of Health of California, in a note dated February 20, 1879, says: "There are no cases of, or deaths from, hydrophobia on the records of the State Board of Health. Personally I know of no cases of the disease in the State." The records of the Health Office in San Francisco furnish no evidence that there has ever been a death from rabies in the city. Is it truthfully stated, then, that climate has no influence over this disease?

It seems to be well proven that rabies prevails more or less in nearly all portions of the world that have been long in communication with the older nations, but that it does not prevail on the American Pacific coast in any animals, brute or human, while at the same time there is every kind of climate from the north frigid, through the temperate and tropic, to temperate and frigid again. Whatever the future may bring, the past gives no record or knowledge of the existence of rabies. Why we are so wonderfully and fortunately favored cannot, perhaps, be positively made out.

It is reasonable to suppose that among the many human beings and animals who are bitten every year by really rabid dogs in the Eastern States and in Europe, some one or more, considering the possibly long period of incubation, must have reached the Pacific coast; yet not one case has been developed, while dogs are numerous and of great variety, from the pure-blooded greyhound to the scurvy mongrel.

Dr. Verity, in an article on this subject which appeared in the Manchester *Courier* of 1876, approaches what may be considered some explanation. He says: "Certain peculiar changes in the system, possibly due to atmospherical influences, act in producing it." If this be true, may not other atmospherical influences act to restrain or annihilate the poison? We have a recognized condition of the atmosphere on the coast, called electrical; and all are aware of the wonderful effects of artificially generated electricity when used in the treatment of nervous and other diseases. A test of the power of the climate of the American Pacific coast to prevent the development of rabies can easily be made, with strong probability of proving a blessing to hundreds of human beings who have no other hope. The period of incubation is so long in many cases, that several dogs known to be badly bitten by a dog known to be suffering with rabies could be sent here under guard, and kept behind prison bars and under observation until the extreme limit was passed. Or a more practicable plan might be to have a number of the many persons who are annually bitten by rabid dogs in the Eastern States and Europe come to this coast, and remain here until the extreme limit of the possibilities was passed, selecting any place from Cape Horn to Behring's Strait—preferably, however, California, where we have a varied climate and all kinds of dogs, and are in close communication with all parts of the world. The patient having a knowledge of these facts, and the support of hope of escape, it might be a power to prevent an attack ; and, if he were attacked, the disease might be so modified by the climate as to render it susceptible of cure.

---

DISSEMINATED TUBERCULOSIS NOT ORIGINATING IN A PRIMARY SOURCE OF INFECTION WITHIN THE BODY. By C. CREIGHTON, M.D.

The author had examined the organ and parts from a number of tuberculous cases, from nine months to fifty years of age, and concluded that there had been no primary seat or focus of disease in the body, with respect to which the tuberculosis might be regarded as a secondary infection. On the other hand, the tubercles on the serous membranes, in the lymphatic glands, in the lungs and in the viscera, were to be taken as all coördinate with respect to the initial infection, which must have been considered to have been a virus introduced into the body from without.

The best analogy for tubercles disseminated through the body was the analogy of primary, secondary, and tertiary syphilis, in which the syphilitic formations, in however various parts they might be situated, and at whatever intervals of time they might appear, were all alike due to a virus introduced from without. The author's views were in accordance with those of Klebs (Virchow's *Archiv.*, vol. xliv, 1868). They were at variance with the opinion of Schüppel (*Lymphdrüisen-Tuberculose*, 1871), who considered that tuberculosis might originate as a primary new formation in the lymphatic glands, and in the serous and synovial membranes. They were equally at variance with the opinion of Rindfleisch, who, while he admitted a primary, secondary, and tertiary tuberculosis, explained that succession as being a subordinate one on the analogy of tumor-infection, which was contrasted with the analogy of the coördinate syphilitic infection, or of any other such infection due to a virus introduced into the body from without.

Professor Virchow (Berlin) criticised the statement of Mr. Treves, that tubercle was not neoplasm, but an inflammatory product. He (Dr. Virchow) did not understand the difference. A neoplasm was a new formation, which had arrived at a certain degree of independency. The formation of new bone on the surface of the old bone was first inflammatory, but, if it grew and formed an exostosis or osteoma, then it was neoplasm. In his opinion, neoplasm was only a conventional term, having no connection with the cause, the origin, or the history of the product. The relations between tubercle and inflammation were manifold. There were forms very common in serous membranes, where the development was at first, and during a long time, simply inflammatory; and it was only after the repetition of certain actions that the development of tubercle in the newly-formed tissues was seen. In some cases, there was a series of capillary excrescences of only fibrous tissues, and in other cases such processes became enlarged at the end, and the formation of tubercle commenced. In other cases, tubercle was developed without any sign of inflammation; that was to say, if the signification of inflammation in the old traditional sense were maintained. Mr. Treves had said that giant-cells were lymph-coagula. When he (Professor Virchow) was a student, all such cells were called "mother-cells." At a later period, he introduced this name of "giant-cells," in order to make a distinction between somewhat large multi-nuclear, with two, three, or four nuclei, and the cells having the characteristic and specific form found in a certain number of cases. It was important to distinguish between multi-nuclear cells and the peculiar giant-cell. In cancer, there would frequently be found large cells with six, eight, or ten nuclei; but the specific contents of the cell were not of the finely granular substance which gave the peculiar appearance to the giant-cell, and it was possible at first sight to distinguish the two forms. This penular form of cell—not the old "mother-cell"—was first distinguished by M. Robin in the marrow of bones, and called by him *myéloplaxè*. The English surgeons applied this term to the history of tumors and neoplasms, and recognized it as characterizing a form of myeloid growth. This was a very penular form of tumor, but it also existed where there was no marrow. Some cases of epulis were composed nearly altogether of those cells, the majority of which proceeded from the surfaces of the maxillary bones. He investigated these penular myeloid cells, and had found them in the placenta and in the peritoneum. M. Robin called them *myéloplaxès*, because he believed that they were not cells. They had a membrane, but, to see it, it was necessary to examine very fresh preparations, such as those just removed in surgical operations. This could be done by applying concentrated fluids, first strong, and afterwards diluted; by which means it became possible to remove the cell-membrane from the surface. He did not know that any coagula could be treated by fluids of different concentration in such a manner that a perfect membrane could be removed. The cells were developed by a regular gradation from simple cells, showing that it must be organic formation, and not coagulum. More developed forms were also characterized by a difference in the granular contents, and which sometimes had a brownish hue. The common multi-nuclear cell had not this peculiar development. He held that giant-cellswere a very peculiar form of regular cell-formation; not common to one single process. He had observed giant-cells in ordinary lymphatic glands long before he and others saw them in scrofulous or tuberculous glands. With regard to Dr. Creighton's communication, he believed that in many cases no caseous or other process existed before the tubercles were formed. He had always maintained that primary eruptions of tubercles might exist without any previous tubercular or caseous mass. He had also seen secondary tubercular deposit at the periphery of cancerous tumors; and hence he had become very cautious in concluding, from the observation of tubercles in the periphery of a tumor, that the origin was also tubercular. He originally believed that the majority of caseous masses found in the suprarenal capsules were tubercular, but possibly that many of those originated in scrofulous inflammation; and that only at a later period tubercle appeared. The secondary eruption might be produced by any noxious substance, or, he would say with Dr. Creighton, by any virus; but this need not come from without. It might be produced in the body itself by any morbid process. He thought that by a multitude of processes a substance could be produced that could infect the neighboring tissue, also the whole body, and produce tubercular eruption. Professor Virchow then referred to the occurrence of bovine tubercle in man, contended for by Dr. Creighton, and handed a copy of the report of the experiments conducted at the Veterinary School in Berlin. It was a matter by no means settled at present. He had never seen the flesh or milk of animals suffering from *perlsucht* cause tubercle in animals fed with it; infection had taken place only by inoculating the tubercular matter itself. He considered that the calcification of the *perlsucht* nodules was a distinguishing characteristic.

M. Béchamp (Lille) directed the attention to the part taken by micro-organisms in the formation of pulmonary tubercle. He thought that the molecular granulations break down, destroy the cells, and take up carbonate and phosphate of lime. They appear under the form of small spheres, or often in that of a figure eight. When placed in a suitable medium, they fructify and give rise to a mycelium and bacteria, this development being accompanied by the disengagement of hydrogen and carbolic acid and the formation of butyric acid.—*The Cincinnati Lancet and Clinic.*

---

THE OPIUM, CHLORAL and HASHISCH HABITS.—Under the title of "Drugs That Enslave," Dr. H. H. Kane, of New York, has produced a small volume (Presley Blakiston, Philadelphia), presenting a very graphic picture of the manner in which the habits of taking opium or morphia, hydrate of chloral, and Indian hemp, are acquired, and containing an accurate description of the injurious effects of these habits upon the system, and the treatment to be employed in breaking them up.

In ministering completely to his natural wants and cravings, man may be said to have several objects in view. His first care is to provide the materials capable of rebuilding the tissues that are constantly being destroyed in the exercise of the different functions of the body ; of these materials, bread, meat and water may be taken as examples. Next, he endeavors to lessen mental uneasiness and anxiety, employing for this purpose various fermented liquors, all of which depend upon the same ingredient—

alcohol—for their intoxicating properties. Finally he strives to multiply and exalt his intellectual and animal pleasures by the use of narcotics, such as tobacco, opium or hemp. It is impossible to find a tribe of people, however savage, that has not discovered in some way, that, for the want of a better word, can be termed instinctive, the art of preparing alcoholic drinks, and that does not use them to produce intoxication, with its attendant pleasures and consequent miseries. Equally universal and instinctive is the use of narcotics. With the exception of tobacco, which is consumed by nearly the whole world, the narcotics resorted to vary greatly in their nature, and, unlike the fermented liquors, owe their soothing effects to very different active principles. The rule is for each nation to use a narcotic of native growth; for instance, in Turkey and China opium is used, in India hashisch, in the Eastern Archipelago betel-nut and betel-pepper, in Peru and Bolivia coca, and so on. While the pleasureable sensations obtained from alcohol and the narcotics explain in part their very extended use, another explanation is to be found in the well-known fact that, when taken in moderation, the consumer is enabled to accomplish his work upon a less amount of food than would otherwise be required; this property is due to their power of diminishing the tissue changes incident to all labor, and the consequent necessity and demand for reparative food.

The effects of food, alcohol and the narcotics depend solely upon the way in which they are employed, whether temporately or intemporately. So natural is it to satisfy the appetite for food, that we are apt to ignore the fact that over-indulgence is as certainly followed by disease as in the abuse of wine or opium. On the other hand, we forget that, of the multitudes who use alcohol and narcotics, very many are not only uninjured, but even benefitted, by their temporate use. In illustration, it is only necessary to refer to the testimony relating to the effects of opium upon the Chinese. On this point, an observer states: "Although the habit of smoking opium is universal among rich and poor, yet they are a powerful, muscular and athletic people, and the lower orders more intelligent and far superior in mental acquirements to those of corresponding rank in our own country

(England);" another: "that the effects of the abuse of the drug do not come frequently under observation." There can be no question, however, about the great tendency to the abuse of both alcohol and narcotics. The sensations produced are so delightful that, having been once experienced, many individuals are unable to resist the temptation to reproduce them. Each indulgence, beside lessening the power of resistance, is followed by a period of depression directly demanding a repetition of the dose, which must also be constantly increased in amount to bring about the desired result. Consequently, what is at first a matter of choice soon becomes a necessity, and the habit of abuse, with its dire consequences, is established.

To limit attention to the narcotics, it is probable that the increase in the habitual over-use of the more powerful substances of this class in our own country does not depend on a simple craving for pleasureable sensations, but, as Dr. Kane suggests, on a desire to be relieved from some long-standing and perhaps constant disagreeable sensation. The increased mental strain attending the struggle for wealth and position, the diminished physical activity, the hurry and turmoil of our lives, together with the influences of climate, have gradually given us bodies in which the nervous element largely predominates.

The distressing manifestations of increased nervousness, particularly pain, are most promptly overcome by narcotics. One of these drugs, usually opium, may be ordered by a physician, the intention being to resort to some more radical treatment as soon as the urgent symptoms have disappeared; the sufferer, though, having once found relief, insists upon a further use of the drug—may even at times feign illness to procure it—and in the end obtains some for himself, and in secret drifts into its habitual misuse. In proof of this statement, it will be found that the particular narcotic and the manner of consumption among *habitués* corresponds exactly with the medical practice of the period in which they live. When opium in substance was prescribed by physicians, people ate opium; when the alkaloid morphia became fashionable, the habit of eating morphia arose, and since the discovery of the hypodermic syringe by Dr. Alexander Wood, of Edinburgh, and its introduction to this country in

1856, by Prof. Fordyce Barker, the habit of injecting morphia subcutaneously has been developed. The chloral habit has demanded attention for the past few years only, the date of the discovery of the drug being comparatively recent; and the hashisch habit is very infrequently met with in this country, simply on account of hemp being so little used as a medicine here.

Where the responsibility for the formation of these habits rests, is a difficult question to decide. A great share of the blame must, of course, be attached to the individual who allows himself to give way to a morbid appetite; but the physician who, knowing the weakness of human nature, places a narcotic at the disposal of a patient, to be used as he deems fit, does a foolish, if not criminal act; still more the druggist who, as far too frequently happens, sells opium or chloral at the simple request of a customer, and without the authority of a physician's prescription.

The opium habit claims a much larger number of victims, is more easily formed, and much more injurious to health than the chloral habit. In proportion to the quantity consumed, the functions of the mind and body are rapidly or slowly undermined, until the *habitué* becomes a mere wreck of his former self; business is neglected; social ties are sundered, and the sole object in his life is the gratification of a passion that he loathes, yet can not overcome. The habit may be broken either by a sudden or a gradual withdrawal of the drug. On account of the suffering occasioned by the first method, and the risk of the production of dangerous symptoms that may continue for many days, the author of the work before us recommends the system of gradual withdrawal, from four to ten days being the period allowed to elapse between the first reduction of the dose and the total suspension. The patients are more successfully treated when removed from their homes and separated from friends who may be induced to secretly supply the drug; baths, electricity and certain medicines are useful as adjuncts, and a very important step in the permanent cure is the removal of the disease that primarily led to the use of opium.

The continued use of hydrate of chloral is not so apt to generate a morbid craving for it as a similar use of either opium or morphia, and the habit, once formed, is usually readily abandoned, although in some instances, like the dipsomaniac's desire for alcohol, the chloral craving, after remaining latent for a long time, unexpectedly returns; under these circumstances, there is considerable difficulty in breaking the habit permanently. Sometimes, particularly when the quantity consumed each day is small, chloral produces no noticeable alteration in the general health. In other cases, the activity of the brain and the whole nervous system is diminished, the functions of digestion, respiration and circulation are interfered with, various eruptions appear upon the skin, and a condition resembling ordinary blood-poisoning may be produced. Unless there is great debility, it is perfectly safe to break this habit by an abrupt withdrawal of the drug; the patient is then soon restored to health by the employment of baths, electricity, and a stimulant and tonic plan of treatment.

Only one instance of the hashisch habit has come under the observation of Dr. Kane, and it is probable that the abuse of this drug is very rare in America.

Dr. Kane's book is adapted more for the professional than the lay reader; still, it contains many points of general interest, and cannot fail to aid in checking an evil that is but too evidently on the increase.—*The American.*

---

NEW YORK AND PHILADELPHIA CLINICAL LECTURES (adapted from the *Denver Tribune* "Primer").—Whom do I see? You see a lecturer describing a case to his class. Do you see the case or the class? Of course you do not; they are both in the lecturer's mind.—*Record.*

